# HIV Spending as a Share of Total Health Expenditure: An Analysis of Regional Variation in a Multi-Country Study

**DOI:** 10.1371/journal.pone.0012997

**Published:** 2010-09-24

**Authors:** Peter Amico, Christian Aran, Carlos Avila

**Affiliations:** 1 Heller School for Social Policy and Management, Brandeis University, Waltham, Massachusetts, United States of America; 2 AIDS Financing and Economics Division, UNAIDS, Geneva, Switzerland; Massey University, New Zealand

## Abstract

**Background:**

HIV has devastated numerous countries in sub-Saharan Africa and is a dominant health force in many other parts of the world. Its undeniable importance is reflected in the establishment of Millennium Development Goal No. 6. Unprecedented amounts of funding have been committed and disbursed over the past two decades. Many have argued that this enormous influx of funding has been detrimental to building stronger health systems in recipient countries. This paper examines the funding share for HIV measured against the total funding for health.

**Methodology/Principal Findings:**

A descriptive analysis of HIV and health expenditures in 2007 from 65 countries was conducted. Comparable data from individual countries was used by applying a consistent definition for HIV expenditures and total health expenditures from NHAs to align them with National AIDS Assessment Reports. In 2007, the total public and international expenditure in LMICs for HIV was 1.6 percent of the total spending on health, while the share in SSA was 19.4 percent. HIV prevalence was six-fold higher in SSA than the next highest region and it is the only region whose share of HIV spending exceeded the burden of HIV DALYs.

**Conclusions/Significance:**

The share of HIV spending across the 65 countries was quite moderate considering that the estimated share of deaths attributable to HIV stood at 3.8 percent and DALYs at 4.4 percent. Several high spending countries are using a large share of their total health spending for HIV health, but these countries are the exception rather than representative of the average SSA country. There is wide variation between regions, but the burden of disease also varies significantly. The percentage of HIV spending is a useful indicator for better understanding health care resources and their allocation patterns.

## Introduction

HIV has had a devastating impact on many countries, particularly those in sub-Saharan Africa (SSA). For example, the life expectancy at birth in Botswana fell from 65 years in 1990 to less than 40 years by 2005 [1]. This is largely attributed to the rise and spread of HIV. Such trends have been similarly observed across numerous other African countries. The world community has responded positively to the HIV threat through investment of funds and recognition of HIV as a global humanitarian crisis. In 2000, global leaders created the Millennium Development Goals (MDG), with goal number six targeting HIV/AIDS, malaria and tuberculosis [2]. In 2005, The Human Development Report concluded that “the HIV/AIDS pandemic has inflicted the single greatest reversal in human development” [3]. Containment of HIV is understood to be a global public good which improves the well-being of all citizens. Developed countries have raised extraordinary funds for controlling this disease, with total funding reaching US$ 15.6 billion in 2008 [4]. These initiatives are responsible for mobilizing the largest amount of funding given to a single disease in history.

One of the most discussed topics accompanying this extraordinary amount of HIV funding is the debate between a silo approach to health financing and a health systems strengthening approach. Many have pointed to the fact that vertical programs, those focused on a specific disease, may be diverting funds away from horizontal programs [5], those interventions that strengthen the entire health system [6]. Advocates of vertical programs point to their service specialization, better accountability and rapid results within weak health systems [7], [8]. Others argue that disease-focused approaches have been extremely successful in both their primary goals as well as in their provision of marginal positive externalities, as demonstrated by programs such as polio eradication [9]. Alternatively, a third argument suggests integrating vertical programs within the existing health system in order to maximize the positive synergies of both the health system and specific programs [10], [11], [12].

As a result of such arguments, there are presently several major donors, including the President's Emergency Plan for AIDS Relief (PEPFAR) [13] and The Global Fund to Fight AIDS, Tuberculosis and Malaria (Global Fund), who are increasing their support of health systems strengthening beyond their disease-specific mandates [8]. In fact, PEPFAR is moving toward prioritization of country ownership of funding flows; PEPFAR's stated goals include to “integrate and coordinate HIV/AIDS programs with broader global health and development programs to maximize impact on health systems” [13]. Marchal *et al.* claim that while health systems strengthening may be a stated goal of some major organizations, their implementation falls short of desired outcomes [14]. One potential solution, similar to PEPFAR's recent goals, involves providing recipient countries with more ownership in the decision-making process [15]. These arguments are highly controversial when it comes to HIV funding, which has escalated in low- and middle-income countries (LMICs) from US$ 292 million [16] in 1996 to over US$ 15.6 billion in 2008 [4], [17].

However, after years of ongoing debate regarding the potential benefits or harms of vertical programs, there remains a paucity of evidence upon which to develop informed policy decisions. This may be partially explained by the difficult nature of conducting rigorous research. There have been some attempts to explicate this relationship between disease-specific funding and health system performance. A few studies have looked at funding flows from the Development Assistance Committee (DAC) and pointed to HIV's increasing percentage [6], [18]. However, this fails to account for the total amount of funding that is spent on health in these countries.

Understanding the share of HIV and total health resources is critical to delineating the aid architecture in LMICs, where the HIV burden often exceeds the available resources. Previous reports argue that countries and donors often allocate more resources to HIV than other health concerns [19]. Others assert that HIV funding in various African countries may in actuality exceed their entire budget for health, thus subverting national priorities [20]. With both donor aid and government resources affected by the global recession [21], it is imperative to achieve an evidence-based understanding of resource allocation in order to maximize health delivery.

This paper seeks to examine funding shares for HIV, especially in SSA, using updated data from the World Health Organization and the Joint United Nations Programme on AIDS (UNAIDS). This allows for investigation of the share of funds allotted uniquely to HIV and other specific diseases at both a global level as well as within specific regions.

The authors' objective is to use the most recent data on domestic spending for total health and HIV, in order to analyze expenditures reported from LMICS and to examine if HIV is receiving a disproportionate share of resources. The paper describes levels and patterns of domestic HIV spending from public and international sources, while taking epidemic types and country income levels into account.

## Methods

A descriptive analysis of HIV and health expenditures in 2006–7 from 65 countries was conducted. All expenditures, by programmatic activity and HIV services, were cross-tabulated by source of financing and stratified by income level. Spending information from public and international sources was analyzed based on the National AIDS Spending Assessment (NASA) methods and classifications [22]. In order to examine HIV funding flows, the authors used two data sets: 1) the 2006 and 2007 National AIDS Spending Assessment and 2) the 2007 data for National Health Accounts (NHA) [23].

NASA is a tool developed by UNAIDS, based on the national health accounts framework. which measures all resources included in a country's national HIV response [22], [23]. The National Health Accounts framework as well as NASA apply standard accounting methods to reconstruct all transactions in a given country, ‘following the money’ from the funding sources to agents and providers and eventually to beneficiary populations.

The NASA financial flows related to health and HIV activities are organized into six areas : 1) financing sources (funding entities that disburse money to agents); 2)agents (entities that receive and pool financial resources, pay for service provision and make programmatic decisions); 3) providers (entities that produce and deliver HIV services); 4) production factors (resources used to produce goods and services); 5) HIV spending categories (goods, services and activities delivered as part of the HIV response); and 6) beneficiary populations (groups targeted by specific programs and activities) [22]. Standardization of all NASA spending categories across countries has been improved through the publication of manuals in English, French, Portuguese, Spanish, Russian and Arabic [22].

The NASA tool, developed by UNAIDS, represents the most ambitious attempt to collect spending information at the national level and to monitor expenditures at the global level [22]. NASA was developed to produce accurate and detailed in-country estimates of the actual expenditures of HIV programs and has been used to report progress on the 2001 Declaration of Commitment from the United Nations General Assembly Special Session on HIV/AIDS (UNGASS). As of 2008, a total of 109 countries had reported domestic spending from international and domestic sources [24].

National Health Accounts measure the various aspects of a nation's health expenditure. It implements a rigorous classification of the types and purposes of all expenditures and of all the actors in the health system, and provides a complete accounting of all spending for health, regardless of the origin, destination, or object of the expenditure [23]. The most reliable NHA data are broken down by the private and government agents who spent health funds. Out-of-pocket (OOP) expenditures and spending on private insurance were excluded in order to match the NASA data, which does not contain private expenditures. The remaining balance was the total amount of spending on health from public and international sources. Due to the difficulty in collecting private expenditure data on health for HIV, only the total public and international expenditures from the NHA and NASA data were used. Additionally, in order to produce meaningful comparisons among countries, all expenditures were put into current 2007 dollars.

Thus, this study used comparable data from individual countries by applying a consistent definition for HIV expenditures and total health expenditures from NHA. The 2007 NASA database provided 71 countries, 15 of which were excluded either because they had low levels of spending on HIV, had a low percentage of representative countries in the region or were high income countries (Sao Tome, Seychelles, UK overseas territories, Palau, St Kitts Nevis, Cuba, Trinidad and Tobago, Egypt, Kuwait, Syria, Fiji, Czech Republic, Hungary, Spain, and the United Kingdom). By taking the 2006 NASA data and applying a regional growth rate based on regression analysis of previous data, 32 additional countries were added, of which 11 were excluded based on the aforementioned criteria (Bahamas, Haiti, Saint Lucia, Cape Verde, Equatorial Guinea, North Sudan, South Sudan, Mongolia, Australia, Algeria, and Switzerland). Within the NASA database, expenditures that were used for health and those that were used for non-health purposes were distinguished. Western and Central Europe (WCE) included a low number of countries with available data, but it was included in the data set in order to capture the few LMICs in the region.

Weighted averages from population-adjusted spending totals for both health and HIV by region were used to calculate the percent of total regional health resources being used for HIV. In SSA, the total HIV spending was biased downward due to the lack of spending data from South Africa, which alone added approximately US$ 1 billion in HIV health spending in 2010 [25]. In order to avoid leaving out this important country a sensitivity analysis was performed, using a conservative estimate of US$ 700 million spent for HIV health in 2007.

Categorization was determined by applying a three-tiered decision-making rubric specific to health spending: 1) “activities whose primary purpose is to restore, improve and maintain health for the nation and for individuals,” consistent with WHO's definition of health [23]; 2) activities that took place within the health system; and 3) actions that were administered by personnel who received compensation from the health sector. The first criterion served as the most important, and the additional criteria were used to clarify the classification if further questions remained. All items not broken down by type or not classified were excluded from this analysis.

Using the above-described rating system, two researchers categorized 136 lines of NASA expenditures independently with 96.3 percent agreement (kappa .875 p<.0001). The remaining five lines of discordance were sent to a third independent party for review. If further questions arose, all three researchers discussed and determined the appropriate classification. Health and HIV spending categories were then matched by using a previously published cross-walk tool [26]. This ratio of health to non-health spending was then applied to total HIV expenditures in LMICs as well as to the individual country spending on HIV to obtain country-specific values for the total HIV spending on health.

For regional analyses, the country-specific proportion of HIV spending for health and total health expenditure were adjusted for population size [27]. The total regional HIV health spending and health expenditures were divided by the size of the population to obtain per capita rates for each region. For the 65 country analysis, all HIV expenditures were totaled and taken out of the aggregate spending on health of those 65 countries to report HIV spending as a share of total health spending.

Countries were classified by region and income level. Economies were ordered according to their Gross National Income (GNI) per capita for the data collection year used, according to the World Bank Atlas Methods [28] and grouped into four categories: low-income (US$ 935 or less); lower middle-income (US$ 936–$3,705); upper middle-income (US$ 3,706–$11,455); and high income (US$ 11,456 or more). Out of the 65 countries, there were 28 low-income countries, 20 lower middle-income countries and 17 upper middle-income countries.

In order to compare various indicators of HIV's global impact, 2004 projections from the Global Burden of Disease Report were used, including disability-adjusted life years (DALYs) and the percent of deaths attributable to HIV, to calculate 2008 predictions [29]. Additionally, estimates of HIV prevalence published in the 2008 UNAIDS Global Report were obtained [24]. These 2008 indicators were used in order to compare a burden of disease measure against the 2007 spending data. Western and Central Europe were left out of this burden of disease analysis because the large number of high-income countries in that region would likely bias these measures in a downward direction. Additionally, the correlation was examined and a regression was performed on HIV spending on health and prevalence in SSA. Prevalence was available for all but two of the study countries in SSA (Kenya, Democratic Republic of Congo) [24].

## Results

Due to the large variation in global HIV spending, five regions were examined separately—sub-Saharan Africa, South East Asia (SEA), Eastern Europe and Central Asia (EECA), Western and Central Europe, and Central and South America (CSA). The study was able to incorporate 67, 45, 75, 16 and 76 percent of the countries from these regions in our data set, respectively. Sub-Saharan Africa countries spent 19.4 percent of their total health expenditures on HIV-related activities, compared to CSA spending of less than 1.1 percent. There were wide variations in spending patterns across regions.

**Figure 1:**
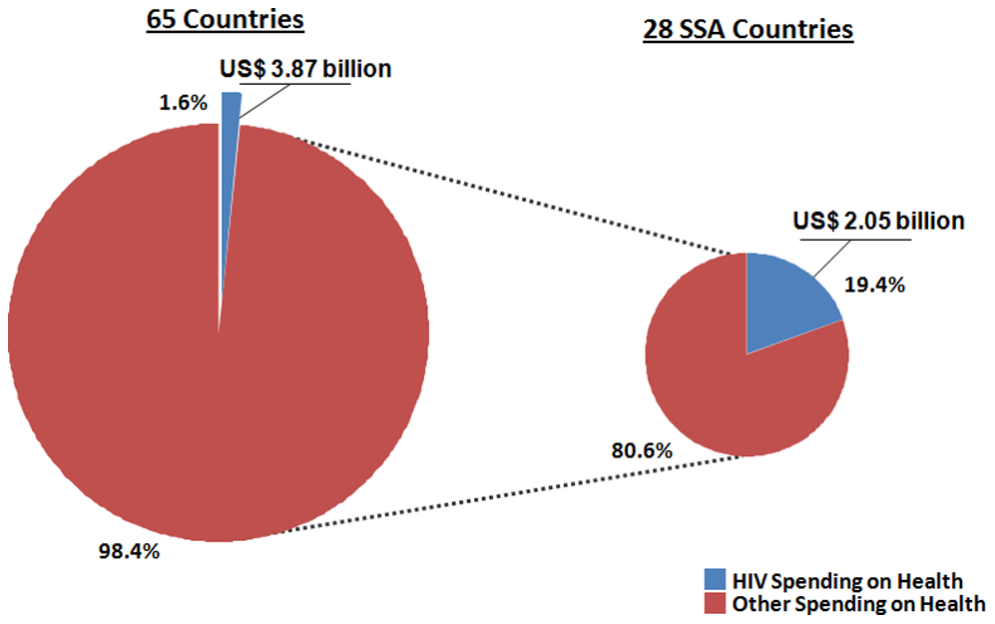
HIV Spending as a Share of Total Health Spending.

Looking at all 65 LMICs included in the analysis, overall HIV spending was overwhelmingly directed to health activities, which comprise 95.1 percent of spending. This represented more than US$3.87 billion spent on HIV health in the 65 LMICs [22]. The remaining 4.9 percent went to activities such as human rights and support for orphans and vulnerable children. After applying the ratio of spending for HIV health, the total public and international expenditure in LMICs for HIV was 1.6 percent of the total spending on health in 2007. Figure 1 shows HIV spending as a share of total health spending in the 65 included countries compared against the share in SSA. The spending ratio for countries from SSA was significantly higher at 19.4 percent, thus predicating the need for regional analyses to further understand this discrepancy.

Table 1 shows a detailed breakdown of the spending across all the included countries. The 28 SSA countries in our study spent US$ 2 billion on HIV health, but their total health expenditure was only US$ 10.56 billion (Table 1). South and South East Asia spent US$ 517 million for HIV health, but their total spending on health of US$ 83.35 billion was more than 8 times the spending in SSA. Spending US$ 1.04 billion, the 13 CSA countries were the other major regional spender of HIV funds, but they spent over US$ 90.37 billion on total health. The 28 SSA countries spent a population-weighted average of US$ 4.08 per capita on HIV health, with the next highest region being CSA at US$ 2.63 per capita. SEA, WCE and SEA spent much less on HIV health, with per capita estimates of US$ 1.34, 0.97 and US$ 0.27 respectively. However, the population-weighted average of total health expenditure per capita was highest in WCE (US$ 413), followed by CSA (US$ 229), EECE (US$ 114), SEA (US$ 43) and SSA (US$ 21). Sub-Saharan Africa spent more per capita than any other region on HIV and less per capita than any other region on health.

**Table 1:** Total HIV Spending, Health Spending, Population, HIV Spending per Capita, Total Health Expenditures per Capita, and the Share of HIV Health Spending.

Region	Year	HIV Hlth. Spending	Tot. Hlth. Spending	Population	HIV/Cap.	THE/Cap.	Pct. HIV
**Sub Saharan Africa (SSA)**							
Angola	2007	47,433,144	$ 1,208,137,245	17,432,462	$ 2.72	$ 69.30	3.9%
Benin	2007	16,625,950	$ 138,738,963	8,514,502	$ 1.95	$ 16.29	12.0%
Botswana	2007	225,640,838	$ 646,084,124	1,907,226	$ 118.31	$ 338.76	34.9%
Burkina Faso	2007	31,410,046	$ 254,145,067	14,192,090	$ 2.21	$ 17.91	12.4%
Burundi	2007	23,839,832	$ 84,601,892	10,274,028	$ 2.32	$ 8.23	28.2%
Cameroon	2007	34,028,774	$ 303,430,563	18,987,350	$ 1.79	$ 15.98	11.2%
Central African Republic	2007	10,036,489	$ 26,661,144	4,277,353	$ 2.35	$ 6.23	37.6%
Chad	2007	8,539,370	$ 194,451,605	10,645,391	$ 0.80	$ 18.27	4.4%
Congo	2007	8,331,154	$ 129,389,566	3,598,672	$ 2.32	$ 35.95	6.4%
Cote d'Ivoire	2007	65,083,758	$ 196,221,558	20,141,042	$ 3.23	$ 9.74	33.2%
Democratic Republic of the Congo	2007	41,400,874	$ 338,130,627	63,321,852	$ 0.65	$ 5.34	12.2%
Gabon	2007	10,141,115	$ 341,681,079	1,430,887	$ 7.09	$ 238.79	3.0%
Gambia	2007	4,520,858	$ 25,880,928	1,594,061	$ 2.84	$ 16.24	17.5%
Ghana	2007	50,208,139	$ 727,597,567	23,159,938	$ 2.17	$ 31.42	6.9%
Guinea-Bissau	2007	2,743,907	$ 14,029,930	1,560,424	$ 1.76	$ 8.99	19.6%
Kenya	2007	387,792,495	$ 638,894,806	37,281,276	$ 10.40	$ 17.14	60.7%
Lesotho	2007	47,667,567	$ 74,042,553	2,020,214	$ 23.60	$ 36.65	64.4%
Mali	2007	37,784,390	$ 218,723,853	11,938,333	$ 3.16	$ 18.32	17.3%
Mauritius	2007	1,190,477	$ 167,117,827	1,290,096	$ 0.92	$ 129.54	0.7%
Mozambique	2007	95,536,946	$ 346,764,475	20,200,475	$ 4.73	$ 17.17	27.6%
Niger	2007	13,750,129	$ 123,241,597	13,863,271	$ 0.99	$ 8.89	11.2%
Nigeria	2007	293,159,644	$ 2,865,597,438	149,055,456	$ 1.97	$ 19.23	10.2%
Rwanda	2007	67,543,637	$ 250,393,827	9,020,701	$ 7.49	$ 27.76	27.0%
Sierra Leone	2007	8,552,918	$ 41,731,554	5,281,803	$ 1.62	$ 7.90	20.5%
Swaziland	2007	31,888,189	$ 135,017,628	1,169,010	$ 27.28	$ 115.50	23.6%
Togo	2007	9,548,938	$ 52,277,189	6,462,731	$ 1.48	$ 8.09	18.3%
Uganda	2007	265,894,160	$ 529,227,579	31,557,498	$ 8.43	$ 16.77	50.2%
Zambia	2007	210,053,166	$ 490,992,403	12,341,879	$ 17.02	$ 39.78	42.8%
**Total**		**2,050,346,903**	**$ 10,563,204,588**	**502,520,021**	**$ 4.08**	**$ 21.02**	**19.4%**
**South East Asia (SEA)**							
Cambodia	2007	53,090,860	$ 204,427,023	14,973,597	$ 3.55	$ 13.65	26.0%
China	2007	100,279,089	$ 65,166,022,589	1,338,070,144	$ 0.07	$ 48.70	0.2%
Indonesia	2007	57,708,323	$ 6,359,737,447	246,797,488	$ 0.23	$ 25.77	0.9%
Lao P.D.R	2007	4,861,487	$ 62,128,802	6,130,845	$ 0.79	$ 10.13	7.8%
Myanmar	2007	30,896,670	$ 56,553,335	56,819,456	$ 0.54	$ 1.00	54.6%
Nepal	2007	13,809,296	$ 249,187,332	25,807,662	$ 0.54	$ 9.66	5.5%
Phillipines	2007	4,739,170	$ 2,161,837,171	90,326,120	$ 0.05	$ 23.93	0.2%
Thailand	2007	192,900,000	$ 6,906,190,585	63,024,352	$ 3.06	$ 109.58	2.8%
Vietnam	2007	59,409,994	$ 2,191,187,287	87,492,048	$ 0.68	$ 25.04	2.7%
**Total**		**$ 517,694,889**	**$ 83,357,271,570**	**1,929,441,712**	**$ 0.27**	**$ 43.20**	**0.6%**
**Eastern Europe Central Asia (EECA)**							
Armenia	2007	2,293,193	$ 210,565,980	3,062,720	$ 0.75	$ 68.75	1.1%
Azerbaijan	2007	2,220,238	$ 422,935,788	8,873,190	$ 0.25	$ 47.66	0.5%
Belarus	2007	17,191,815	$ 2,350,801,732	9,899,022	$ 1.74	$ 237.48	0.7%
Georgia	2007	6,738,971	$ 229,272,673	4,351,759	$ 1.55	$ 52.69	2.9%
Kazakstan	2007	17,340,373	$ 2,593,020,220	16,150,466	$ 1.07	$ 160.55	0.7%
Kyrgyzstan	2007	10,594,974	$ 143,186,758	5,443,675	$ 1.95	$ 26.30	7.4%
Republic of Moldova	2007	8,185,739	$ 235,531,558	3,961,192	$ 2.07	$ 59.46	3.5%
Tajikistan	2007	5,113,815	$ 59,433,551	6,808,692	$ 0.75	$ 8.73	8.6%
Ukraine	2007	72,539,355	$ 5,845,343,798	47,665,032	$ 1.52	$ 122.63	1.2%
**Total**		**$ 142,218,474**	**$ 12,090,092,057**	**106,215,748**	**$ 1.34**	**$ 113.83**	**1.2%**
**Western and Central Europe (WCE)**							
Bulgaria	2007	6,520,255	$ 1,819,152,063	7,811,891	$ 0.83	$ 232.87	0.4%
Croatia	2007	8,907,939	$ 3,893,841,620	4,400,000	$ 2.02	$ 884.96	0.2%
Latvia	2007	6,943,584	$ 1,036,168,133	2,187,549	$ 3.17	$ 473.67	0.7%
Montenegro	2007	1,468,956	$ 257,974,244	625,000	$ 2.35	$ 412.76	0.6%
Poland	2007	41,154,420	$ 20,512,787,004	38,625,876	$ 1.07	$ 531.06	0.2%
Turkey	2007	55,745,494	$ 24,023,076,923	71,158,647	$ 0.78	$ 337.60	0.2%
**Total**		**$ 120,740,648**	**$ 51,542,999,987**	**124,808,963**	**$ 0.97**	**$ 412.98**	**0.2%**
**Central and South America (CSA)**							
Argentina	2007	201,133,620	$ 13,981,548,387	39,627,972	$ 5.08	$ 352.82	1.4%
Bolivia	2007	3,130,254	$ 462,091,726	9,798,686	$ 0.32	$ 47.16	0.7%
Brazil	2007	554,330,346	$ 49,064,102,564	195,902,528	$ 2.83	$ 250.45	1.1%
Colombia	2007	68,096,518	$ 10,607,841,110	45,953,956	$ 1.48	$ 230.84	0.6%
Costa Rica	2007	13,844,314	$ 1,587,316,403	4,608,883	$ 3.00	$ 344.40	0.9%
Ecuador	2007	7,378,944	$ 1,364,000,000	13,900,130	$ 0.53	$ 98.13	0.5%
El Salvador	2007	37,661,693	$ 739,300,000	6,119,093	$ 6.15	$ 120.82	5.1%
Honduras	2007	17,621,759	$ 505,873,016	7,586,580	$ 2.32	$ 66.68	3.5%
Panama	2007	17,453,947	$ 855,200,000	3,464,820	$ 5.04	$ 246.82	2.0%
Paraguay	2007	2,221,986	$ 302,543,495	6,353,777	$ 0.35	$ 47.62	0.7%
Peru	2007	30,340,512	$ 2,744,141,755	29,386,596	$ 1.03	$ 93.38	1.1%
Uruguay	2007	6,687,997	$ 1,405,363,471	3,397,766	$ 1.97	$ 413.61	0.5%
Venezuala	2007	78,868,467	$ 6,753,636,207	28,267,160	$ 2.79	$ 238.92	1.2%
**Total**		**$ 1,038,770,358**	**$ 90,372,958,134**	**394,367,947**	**$ 2.63**	**$ 229.16**	**1.1%**

**Figure 2:**
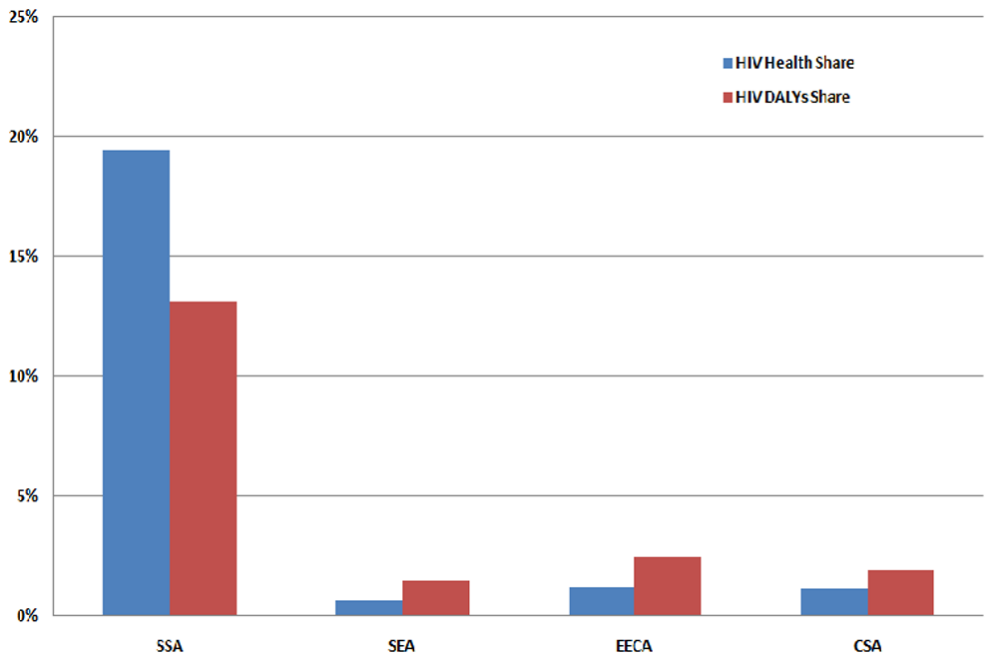
Percent Share of HIV Spending and HIV DALYS by Region.

The total spent on HIV in SSA countries, excluding South Africa, was US$ 2.05 billion, accounting for 19.4 percent of global HIV spending. The total expenditure on health in the 28 included SSA countries was only US$ 10.5 billion, accounting for approximately 2.6 percent of global health spending. There is a high prioritization of HIV in overall health spending in SSA and when looked at through the lens of morbidity and mortality attributable to HIV in 2008 [27], the proportion of funding for HIV in SSA is higher than the burden of disease (Figure 2). However, in the other regions, this is not the case.

Figure 2 illustrates the share of expenditures devoted to HIV spending and HIV DALYs by region. HIV prevalence in SSA is six-fold higher than the global prevalence; DALYs due to HIV in SSA are more than 5 times that of the EECA, which has the next highest value, and SSA's percent of deaths attributable to HIV is almost 10 times greater than CSA, which is the region with the next highest rate [27]. However, the share of spending for HIV only exceeds the share of HIV DALYs in SSA.

Correlation of HIV spending on health with the HIV prevalence of the SSA countries was 0.33 (*p* = .10). The naïve model, including HIV spending and prevalence, showed that a one percent increase in prevalence led to a US$ 3,738,545 (p = .10) increase in HIV spending; however, this model only explained 10 percent of the variation. In order to account for omitted variable bias, total health spending, foreign direct investment (FDI), population size and GDP were included in the regression model. Table 2 shows the different models and their significance levels. With the inclusion of these additional variables into the model, a one unit increase in HIV prevalence led to an increase in HIV spending of US$ 3,964,055 (*p*<0.05) holding total health spending, FDI, GDP and population size constant. The r-squared value of this model was 0.74; thus, the model explained almost three-quarters of the variation in health spending.

**Table 2:** HIV Prevalence as a Predictor of HIV Spending.

Variable	model1	model2	model3	model4	model5
**HIV Health Spending**					
**Prevalence**	3,738,545	3,964,055	4,004,986	4,433,100	3,885,668
**Health Spending**		.102***	.0838*	0.0535	.246**
**FDI**			0.0111	0.00326	−0.00452
**Population**				0.966	4.14*
**GDP**					−.00333**
**Constant**	39,216,214	109,965	276,905	−1,615,432	−22,075,285
**R^2^**	0.108	0.586	0.598	0.607	0.739

Legend: * p<0.05; ** p<0.01; *** p<0.001.

A sizeable divergence between the highest and lowest country-specific values for spending within SSA was apparent. Several countries, including Zambia, Uganda, Kenya, and Lesotho spent a significant portion of their total health spending on HIV, ranging from 42–64 percent (Table 1). More specifically, Kenya and Lesotho were the highest spenders, allocating more than 60 percent of their health spending to HIV. Botswana spent almost US$ 118 per capita on HIV health compared to the average of US$ 9.34 in the rest of SSA. However, they also had an HIV prevalence of 23.9 percent in 2007. While there are high spenders, it seems that spending is often in proportion to the overall burden of HIV, as was suggested by the regression of HIV spending and prevalence. However, a few countries such as Burundi, Niger, and Rwanda spent relatively high amounts compared to their low burden of HIV. Burundi spent 28 percent of their health spending while having a prevalence of only 2 percent. Most countries follow rational spending patterns, while a few outliers' spending may be reliant on other unknown factors.

## Discussion

The share of overall health spending allocated to HIV reached 1.6 percent in 2007. This is quite moderate considering that the estimated share of deaths attributable to HIV stood at 3.8 percent and DALYs at 4.4 percent [27]. However, the share of spending for HIV varies significantly by region. In SSA, the 28 included countries spent 19.4 percent of their total health spending on HIV, while EECA spent 1.2 percent and CSA spent 1.1 percent. This variation between regions is possibly due to differences in the HIV-related burden of disease. Other explanations include factors such as political will of the government or prioritization of HIV by donors. The percentage of total spending on HIV only exceeds the percent burden of HIV deaths and HIV DALYs in SSA. Globally, there seems to be a shortage of spending on HIV, with higher spending patterns in SSA. In fact, if the unit costs of treating HIV are significantly higher than other diseases, this could indicate dramatic under-spending on HIV globally. On the other hand, if diseases like malaria generate even worse outcomes in terms of total deaths or DALYs than HIV, then perhaps less money should be spent at the margin on HIV and more transferred to malaria. This should be the subject of future study.

Critics may point to high spending shares on HIV health as evidence of the over-prioritization of HIV in SSA. However, the claim that HIV is receiving a disproportionate share of global health resources for HIV health may be invalidated when interpreted in light of burden of disease indicators—HIV DALYs, percentage of HIV deaths and HIV prevalence. Moreover, these indicators may not have a one-to-one correlation with HIV spending due to the fact that these indicators are better suited to deal with the cost of HIV management related to morbidity and mortality [30] and do not reflect population prevention needs. Within the region, the countries with higher HIV prevalence spent more than those countries that had a lower HIV prevalence. This finding seems reasonable and it may also point to the need to invest in preventive programs.

Several high spending countries are using a large share of their total health spending for HIV health, but these countries are the exception rather than representative of the average SSA country. The NASA and NHA data provide a useful starting point in trying to understand the effect of HIV funding, but these descriptive data alone cannot definitively answer the causal question of whether HIV funding is hurting the health system. Donors and researchers have tried to assess the impact of HIV funding, but with few successes. Direct evidence that mortality and morbidity have dropped at the country level due to HIV funding is lacking.

There is considerable variation in overall spending among regions. The region with the highest spending was SSA, which spent over 19 percent of their total health spending on HIV health. Inclusion of conservative estimates for South Africa decreases this value to 13 percent. This is mostly due to the fact that South Africa spent US$ 10.72 billion on health, more than the 28 included countries combined. When looking at the included countries in SSA it was found that prevalence and population size predicted over 58 percent of the HIV health expenditures. This is clear evidence of rational funding based on these countries' overall needs as they were related to the burden of disease due to HIV. While there are outlier countries such as Burundi, Rwanda and Niger, the overall spending patterns reflect an evidence-based allocation of funding in these countries.

While the share of spending for HIV is high in SSA compared to other regions, it is certainly not overwhelming the majority of health budgets as is often claimed. However, a few countries, including Zambia, Uganda, Kenya, and Lesotho are spending more than 40 percent of their health budgets on HIV/AIDS. Such a financing arrangement lacks sustainability due to disproportionate spending on a single disease process. However, it is not clear if these are accurate measures, artifacts of under-reported total health spending, or miscommunication between HIV spending and health spending reporting mechanisms. If this imbalance reflects reality, other health services are likely suffering. One must also consider that this large share of health spending may be part of different health systems strengthening initiatives. If this is the case, such an imbalance in funding is less concerning because HIV funds would be simultaneously bolstering the overall health system. On the whole, most countries are spending rational amounts based on the impacts of HIV and the claims that SSA is over-spending seem to be unsubstantiated except in a few select countries.

Considerable opportunities remain for additional research on this topic. Further analysis of country-specific allocation of funds should be undertaken in order to understand the possible mismatch between HIV health spending and overall health spending. Examination of donors' claims that a large proportion of their funds support health systems strengthening activities would be of particular interest. For example, PEPFAR claims that only 48 percent of their 2008 funding went to treatment, with the remainder supporting prevention and care [31]. Similarly, the Global Fund claims that it has helped 4.9 million orphans, provided voluntary counseling and testing (VCT) to 46 million people and trained 7.6 million people to provide care for HIV/AIDS, TB and malaria [32]. And the World Bank's Multi-Country HIV/AIDS Programme (MAP) has stated that one of the four areas of focus in SSA was “strengthening health systems,” which includes training staff, building infrastructure and improving supply chains for drugs and testing kits [33].

Health systems strengthening is clearly a stated priority of donors and a significant portion of spending undoubtedly benefits the surrounding health system in recipient countries. But it is difficult to determine the extent to which VCT, blood screening, prevention of mother-to-child transmission, infrastructure and improved supply chains provide secondary benefits. The literature is still lacking in evidence which quantifies the effects of donor funding.

While this study undertook a novel means of quantifying and categorizing health spending, it faced several limitations. First, the lack of NASA data regarding private expenditures prohibited assessment of individual-level spending on HIV health. The NHA classification of agents is not specific enough to include public and international funding due to some of the broad categories. In order to address this limitation, NHA data that were clearly private expenditures, OOP and private insurance, were eliminated from analyses; the remaining expenditures were clearly public or international. This distinction ensured comparability between NASA and NHA data. However, private expenditures may be a substitute for public expenditures which could bias the analysis; but, these data are not available for HIV spending in the NASA database.

The regression analysis and correlations showed a significant effect between the levels of HIV spending and prevalence, but this analysis may be limited due to the small sample size. Also, the data used here are descriptive, which may help to explain how resources are being used both across regions and globally; however, they cannot be used to make claims of causality. In order to better understand the potential positive or negative synergies of HIV programs, randomized control trials or rigorous quasi-experimental studies need to be conducted.

This paper is unable to solve the debate around vertical programs or elucidate the impact of donor funding for HIV on the overall health system. However, the total spending on HIV as a share of health expenditures regionally is a good indicator to better understand HIV spending patterns. It offers evidence which suggests appropriate allocation of funds according to disease burden in most SSA countries. A few countries demonstrated spending profiles which fail to align with their country-specific burden of disease and should be the subject of further study.
